# The developmental and environmental regulation of gravitropic setpoint angle in Arabidopsis and bean

**DOI:** 10.1038/srep42664

**Published:** 2017-03-03

**Authors:** Suruchi Roychoudhry, Martin Kieffer, Marta Del Bianco, Che-Yang Liao, Dolf Weijers, Stefan Kepinski

**Affiliations:** 1 Centre for Plant Sciences, University of Leeds, Leeds, UK; 2 Laboratory of Biochemistry, Wageningen University, Wageningen, The Netherlands

## Abstract

Root and shoot branches are major determinants of plant form and critical for the effective capture of resources below and above ground. These branches are often maintained at specific angles with respect to gravity, known as gravitropic set point angles (GSAs). We have previously shown that the mechanism permitting the maintenance of non-vertical GSAs is highly auxin-dependent and here we investigate the developmental and environmental regulation of root and shoot branch GSA. We show that nitrogen and phosphorous deficiency have opposing, auxin signalling-dependent effects on lateral root GSA in Arabidopsis: while low nitrate induces less vertical lateral root GSA, phosphate deficiency results in a more vertical lateral root growth angle, a finding that contrasts with the previously reported growth angle response of bean adventitious roots. We find that this root-class-specific discrepancy in GSA response to low phosphorus is mirrored by similar differences in growth angle response to auxin treatment between these root types. Finally we show that both shaded, low red/far-red light conditions and high temperature induce more vertical growth in Arabidopsis shoot branches. We discuss the significance of these findings in the context of efforts to improve crop performance via the manipulation of root and shoot branch growth angle.

The growth angle of lateral root and shoot branches is a central component of the overall architecture of the plant. Almost without exception, branches grow non-vertically for at least a portion of their development, a key adaptation enhancing the uptake of water and nutrients below ground and the harvesting of light above. Importantly, the growth angles of many branches are set and maintained with respect to gravity and not relative to the main or parent axis per se. In this case the organ is said to be maintaining a gravitropic setpoint angle or GSA. Previous studies demonstrated very clearly that GSAs are developmentally-regulated^1^,^2^,^3^,^4^ but that the molecular mechanisms controlling the setting and maintenance of GSAs are only now beginning to become understood. Recently, a prominent role for auxin transport and response in the regulation of lateral organ GSA has been described^5^,^6^,^7^. It has been shown, for example, that treatment with exogenous auxins cause lateral roots in Arabidopsis to grow with a more vertical GSA^6^,^7^. These effects of auxin on GSA control were shown to apply to other species including rice and common bean^6^.

Two recent studies also reported that the different phases of non-vertical growth of lateral roots following their emergence from the primary root are associated with distinct patterns of expression of the auxin efflux carriers PIN3, PIN4, and PIN7. This work showed that PIN4 and PIN7 are absent or very lowly expressed in the columella cells of less vertical young lateral roots, while in the older, more vertical lateral roots PIN4 and PIN7 are upregulated while PIN3 expression diminishes^5^,^7^. It has also been shown that *pin* mutants have altered GSA phenotypes^4^,^7^. Based on these observations, Ruiz-Rosquete *et al*. went on to propose a model in which this characteristic spatio-temporal pattern of PIN protein expression in developing lateral roots underpins their gravitropic response and hence is an important component of growth angle control. Finally, Roychoudhry *et al*. demonstrated that non-vertical GSAs are maintained by means of an antigravitropic offset (AGO) mechanism that counteracts graviresponse in lateral root and shoot branches such that specific growth angles can be maintained^6^. This work showed that like gravitropic response, AGO activity is dependent on auxin transport suggesting a model in which GSAs are maintained by the interaction of two opposing auxin fluxes, which when balanced, determine the angle of growth of the branch^6^. Importantly, the magnitude of the AGO is regulated by transcriptional responses to auxin via the TIR1/AFB-Aux/IAA-ARF system of auxin receptors and transcriptional regulators: lateral roots and shoots of Arabidopsis mutants having higher auxin levels (*yucca1-1d, yuc1D*) or predicted higher levels of auxin response (*arf10-3 arf16-2, axr3-10, arf10-3 arf16-2 axr3-10*) were more vertical than those of the wild-type. In contrast lateral branches of mutants with lower levels of auxin (*wei8 tar2*) or reduced auxin response (*tir1-1, afb4-2 afb5-5*) have less vertical GSAs^6^. These genetic data are consistent with the reported effects of exogenous auxin on lateral root growth angle.

The importance of non-vertical growth for resource capture prompted us to consider the effects of variation in environmental conditions on GSA control. Environmental signals such as light, temperature and nutrient availability have profound effects on plant architecture. For example, in Arabidopsis, a reduction in the ratio of red:far red (R:FR) light leads to a marked increase in hypocotyl and petiole elongation and a corresponding reduction in cotyledon and leaf expansion^8^,^9^. These processes, which form part of the plant’s response to shading by neighbouring plants, require coordinated regulation of distinct developmental processes such as cell proliferation, cell differentiation and direction of cell expansion, all of which are dependent on the action of phytohormones including auxin^10^,^11^. Further studies have also described the effect of high temperature on Arabidopsis plants^12^,^13^,^14^. These studies showed that growing Arabidopsis at elevated temperature of 29 °C leads to accelerated development, bolting, and flowering. In young seedlings grown at 29 °C, the most noticeable effect was a drastic increase in hypocotyl and petiole length relative to control plants and again, these effects were shown to be auxin-dependent^12^,^13^,^14^.

Below ground, changes in the levels and availability of water and nutrients such as nitrate, phosphate, sulphur and iron act as signals that can have a profound impact on root system architecture (RSA) by modulating processes such as root hair growth, primary root elongation and lateral root growth^15^,^16^,^17^,^18^. In Arabidopsis, phosphate deficiency induces a suite of changes in RSA such as shortening of the primary root, elongation of lateral roots and roots hairs^19^,^20^,^21^. In contrast, uniformly high nitrate reduces lateral root elongation, whereas in plants grown on low concentrations of nitrate, exposure of a section of the primary root to high nitrate concentrations leads to local increased lateral root elongation and density^22^,^23^.

Given that auxin response is implicated in the transduction of so many environmental signals and is also central to the regulation of lateral organ GSA, we set out to explore the effect of environmental variation on GSA control and the integration with endogenous developmental programmes. Here we show that in standard, nutrient replete conditions, the more vertical GSA of older lateral roots is associated with increased auxin levels and auxin signalling. Consistent with this finding, and confirming a previous study^24^, we show that low phosphorous levels induce more vertical GSAs in Arabidopsis lateral roots. In contrast, in low nitrogen conditions lateral roots grow at less vertical GSAs. We show that these GSA responses to phosphorous and nitrogen status require TIR1/AFB-mediated auxin signalling. Interestingly, the response of Arabidopsis lateral roots to low phosphate is entirely opposite to growth angle changes reported for adventitious or basal roots in bean. To address this apparent discrepancy we also examine the response of bean basal roots to phosphorous deficiency and auxin. We show that growth angle changes in these roots in response to auxin are dose-dependent; higher auxin concentrations (90–100 nM IAA) induce more vertical growth in basal roots while lower concentrations (50–70 nM IAA) cause basal roots to grow at less vertical GSAs. Finally, we show that under simulated vegetational shade and at high temperature, Arabidopsis shoot branches shift to more a vertical GSA.

## Results

### Developmental regulation of Arabidopsis lateral root GSA

**Figure 1:**
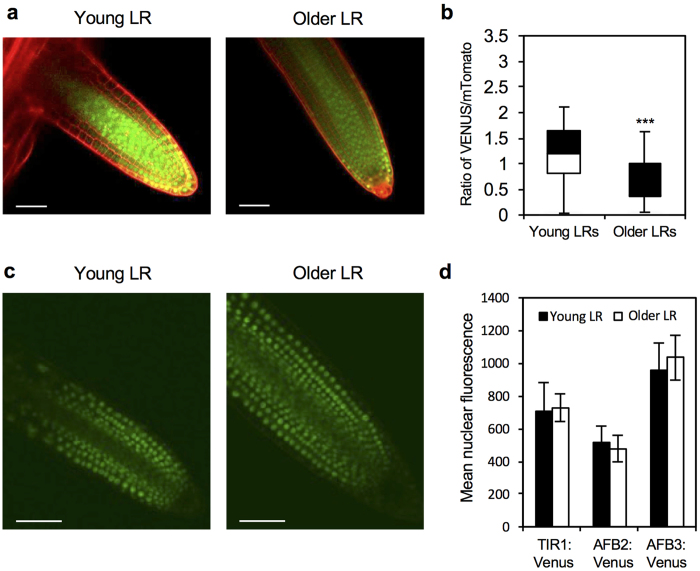
Variation in auxin and auxin signalling input in Arabidopsis lateral roots of different ages. (**a**) Comparison of the expression of DII-Venus in young emerging (<1 mm) lateral roots (left) and older (3–5 mm) lateral roots. Scale bar = 50 μM. (**b**) Ratio of the geometric means of Venus/mTomato signal in epidermal cells of young and old lateral roots of the reporter R2D2 (p = 6.0953 × 10^−12^). (**c**) *pTIR1::TIR1:* Venus expression in young (<1 mm) and older (3–5 mm) lateral roots (as quantified in [d]). Scale bar = 30 μM. (**d**) No significant differences (p > 0.05) in mean nuclear fluorescence observed in lateral roots of different ages in the TIR1/AFB translational marker lines. Bars represent standard error of the mean (s.e.m.).

In Arabidopsis, lateral roots emerge from the pericycle cell layer in a near horizontal orientation and gradually transition towards a more vertical growth angle, eventually attaining a near vertical orientation^3^,^6^,^7^. Importantly, the non-vertical growth phase of an Arabidopsis lateral root does not represent a lack of gravitropic competence but rather the active maintenance of a growth angle that is characteristic of its developmental stage^1^,^3^,^6^. The more vertical branch GSA observed in response to treatment with exogenous auxin and in mutants with higher auxin levels led us to examine the possibility that the observed change in GSA as lateral roots grow out could be attributable to increases in endogenous auxin concentration in the root apex. We used the Aux/IAA-based auxin signalling input reporters DII-Venus and R2D2^25^,^26^ in conjunction with translational reporters for the TIR1/AFB auxin co-receptor proteins TIR1, AFB2, and AFB3 to explore this possibility. Both DII-Venus and R2D2 fluorescent markers are destabilized by auxin in a concentration-dependent manner^25^,^26^. In lateral roots, DII-Venus was expressed at higher levels in young emerging lateral roots (<1 mm) compared to older elongating roots (3–5 mm) indicative of higher levels of auxin in older lateral roots (Fig. 1a). This result was confirmed in the quantitative ratiometric analysis of the R2D2 reporter, which showed that the ratio of auxin-destabilizable Venus signal to auxin-insensitive mTomato was significantly lower in older lateral roots than in young emerging ones (Fig. 1b) (p < 0.001, Student’s t-test). The destabilization of Aux/IAA-based signalling reporters depends upon the presence of TIR1/AFB co-receptor proteins^25^,^26^. To confirm that the observed patterns of DII-Venus and R2D2 expression in lateral roots were not attributable to differences in the expression of TIR1/AFB auxin co-receptor proteins during lateral root development we constructed translational reporters for TIR1, AFB2 and AFB3. Analysis of *pTIR1::gTIR1:Venus, pAFB2::gAFB2:Venus*^14^, and *pAFB3::gAFB3:Venus* reporters revealed that co-receptor levels were not significantly different in lateral roots of different ages (Figs 1c,d and S1). These data indicate that the more vertical GSA orientations in older lateral roots is likely attributable to increases in auxin concentration rather than differences in sensitivity to auxin at the level of TIR1/AFB co-receptor proteins.

### Effects of phosphate and nitrate on lateral root GSA

**Figure 2:**
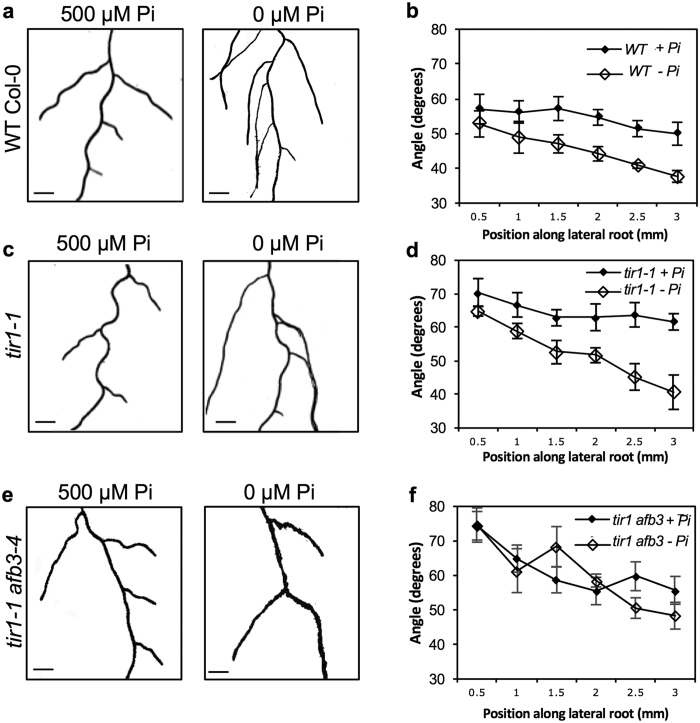
Effect of phosphate deficiency on lateral root GSA in Arabidopsis. Phosphorus deprivation induces a more vertical GSA phenotype in Col-0 lateral roots (**a,b**) as well as in *tir1-1* mutant seedlings (**c,d**), but not in *tir1-1 afb3-4* double mutants (**e,f**). Scale bar = 5 mm. Bars represent s.e.m. from three independent experiments with 10 seedlings each.

**Figure 3:**
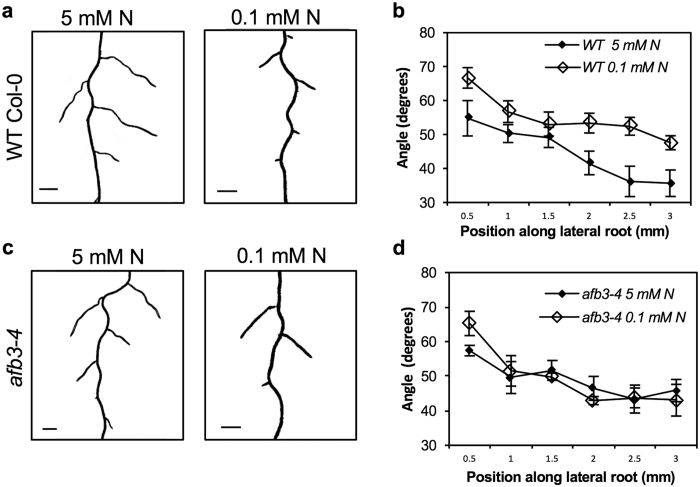
Effect of nitrate deficiency on lateral root GSA in Arabidopsis. Nitrogen deprivation induces a less vertical GSA growth habit in lateral roots of wild-type Col-0 (**a,b**). Lateral roots of *afb3-4* seedlings do not respond to nitrogen deprivation (**c,d**). Scale bar = 5 mm. Bars represent s.e.m. from three independent experiments with 10 seedlings each.

To begin to explore the environmental regulation of lateral branch GSA we first examined the effects of nutrient deficiency on lateral root GSA in Arabidopsis. Nitrogen and phosphorus deficiency had largely contrasting effects on Arabidopsis root system architecture with seedlings growing on low phosphate medium producing more lateral roots while those grown under low nitrate conditions having a reduced lateral root density^19^,^20^,^21^,^27^,^28^ (data not shown). Consistent with previous studies^24^, the GSA of lateral roots grown in phosphorus-deficient conditions was more vertical than those grown in phosphorus-replete conditions (Fig. 2a,b). In contrast, the GSA of lateral roots of nitrogen-deprived seedlings was significantly less vertical than those of plants growing on high nitrogen medium (Fig. 3a,b).

It has previously been shown that low phosphate conditions lead to a significant increase in the expression levels of the auxin receptor *TIR1* in Arabidopsis^20^. Since TIR1/AFB-mediated signalling is known to regulate root branch GSA^6^,^7^ we sought to evaluate the possibility that the reported increase in TIR1/AFB levels may contribute to the more vertical lateral root GSA observed under low phosphorous conditions. In accordance with previous work^20^, quantitative RT-PCR analysis showed that TIR1 expression increased approximately 2.5 fold in roots of wild-type Col-0 grown in phosphorus-deficient conditions (Fig. S2a). We also assessed the effect of phosphate deficiency on the expression of *TIR1* and *AFB1, AFB2, and AFB3* using transcriptional and translational reporter fusions to GUS and Venus respectively^29^. Under phosphorus-replete conditions, we found that *pTIR1::GUS, pAFB1::GUS* and *pAFB3::GUS* reporters were expressed in lateral roots, while *pAFB2::GUS* was largely absent (Fig. S2c and data not shown). Additionally, *pTIR1::GUS* and *pAFB3::GUS* reporter expression in lateral roots increased dramatically under phosphorus deprivation, while *pAFB1:GUS* expression levels remained uniformly high (Fig. S2b and data not shown). Consistent with these data, TIR1:Venus and AFB3:Venus levels in the translational reporters also increased significantly under phosphate-deficient conditions, albeit to a lesser extent than observed at the transcriptional level (Fig. S2b). We therefore decided to analyse the lateral root GSA of *tir1-1, afb1-3 and afb3-4* loss of function mutant seedlings in response to phosphorus deprivation. Consistent with previous work^6^, lateral roots of *tir1-1* and *afb3-4* seedlings grew at less vertical GSAs under phosphorous replete conditions (Figs 2c,d and S3). On low phosphate media, the lateral root GSAs of all three *tir1/afb* single mutant seedlings were shifted towards the vertical to a similar extent to that observed in wild-type plants (Figs 2c,d and S3). This contrasts with the almost complete inhibition of the low phosphorus effect on lateral root number in the *tir1-1* mutant^20^. Given that low phosphate conditions lead to significant increases in transcript and protein levels of both *TIR1* and *AFB3*, we examined the lateral root GSA phenotype of the *tir1 afb3* double mutant. Under phosphate-replete conditions, lateral roots of *tir1 afb3* adopted more horizontal growth angles but showed no change in GSA in response to phosphate deficiency (Fig. 2e,f). As phosphate deficient conditions do not seem to increase auxin levels in the root^20^,^30^, these data suggest that the low phosphorus effect on lateral root GSA is mediated by redundant activity among the TIR1/AFB proteins and principally, TIR1 and AFB3.

Auxin-mediated root architectural changes due to nitrogen starvation have been previously linked to a signalling pathway involving the auxin receptor AFB3^18^. We therefore tested the effect of nitrogen deprivation on the *afb3-4* loss-of-function mutant. Under control conditions (using ATS medium as opposed to Johnson’s medium for the phosphorus studies above) the lateral root GSA of *afb3-4* seedlings was not significantly different to that of wild-type Col-0 (Fig. 3a–d). Interestingly, *afb3-4* mutants did not display the less vertical lateral root GSA phenotype associated with nitrogen deficiency (Fig. 3c,d), suggesting that unlike phosphate starvation, the modulation of lateral root GSA by nitrogen signalling most likely occurs through an AFB3-specific pathway.

### Effects of phosphate deficiency and auxin treatment on basal root GSA in bean

**Figure 4:**
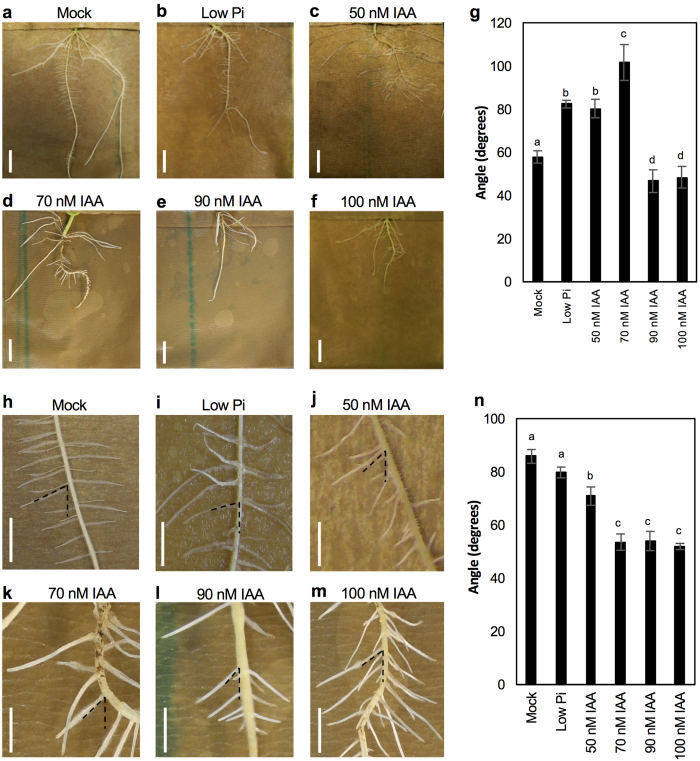
Effect of phosphate deficiency on basal and lateral root GSA in bean. Phosphorus deprivation and treatment with low concentrations of auxin (50–70 nM IAA) cause more horizontal growth of basal roots of bean (**a–d,g**), whereas higher concentrations of auxin (90–100 nM IAA) leads to a more vertical basal root growth angle (**e–g**). p = 6.21 × 10^−12^, one way ANOVA. Tukey’s HSD posthoc tests showed p-values of 0.009, 0.006, 0.001, 0.013 and 0.023 between mock and low phosphorus, 50 nM IAA, 70 nM IAA, 90 nM IAA and 100 nM IAA respectively. In contrast to basal roots, lateral roots in bean adopt a more vertical growth angle upon treatment with increasing concentrations of IAA (**h,j–n**). Low Pi does does not influence lateral root growth angle in bean (**h,i,n**). p = 1.25 × 10^−14^, one way ANOVA. Tukey’s HSD posthoc tests gave p-values of 0.619, 0.004, 0.001, 0.001 and 0.001 between mock and low phosphorus, 50 nM IAA, 70 nM IAA, 90 nM IAA and 100 nM IAA respectively. Scale bar = 1 cm (**a–f**), 0.5 cm (**h–m**). Bars represent s.e.m. of 10–12 roots each from three independent experiments.

Phosphorus deficiency has been shown to cause a shift in the growth angle of basal (adventitious) roots in bean towards a more horizontal orientation^31^. This contrasts with the more vertical GSA response to low phosphorus observed in Arabidopsis lateral roots^24^ (Fig. 2). Because of the central role for auxin in the regulation of lateral branch GSA, we decided to test the effect of phosphate deprivation and increasing auxin concentrations on bean basal roots. Confirming previous reports^31^, growth at low phosphate levels resulted in a less vertical growth angle in basal roots of bean (Fig. 4a,b,g). Treatment with low concentrations of auxin (50–70 nM) resulted in a similar phenotype: a shift towards a less vertical growth angle (Fig. 4a,c,d,g). Interestingly, treatment with a higher concentration of auxin (90–100 nM) caused a significant shift in basal root growth angle towards a more vertical orientation, similar to that reported for Arabidopsis lateral roots^6^,^7^ (Fig. 4a,e,f,g) suggesting a more complex, dose-dependent regulation of GSA in adventitious roots in bean. In contrast to basal roots, lateral root growth angle in bean also shifted towards a more vertical orientation upon treatment with increasing concentrations of IAA^6^, in a dose-dependent manner (Fig. 4h–n), indicative of a differential sensitivity to auxin between bean lateral and adventitious roots. Interestingly, low phosphorous did not influence lateral root angles in bean. Taken together, this suggests that the low phosphorous-dependent increase in global auxin sensitivity in bean lateral roots is not sufficient to affect their growth angle.

### Effect of light and temperature regime on shoot GSA

**Figure 5:**
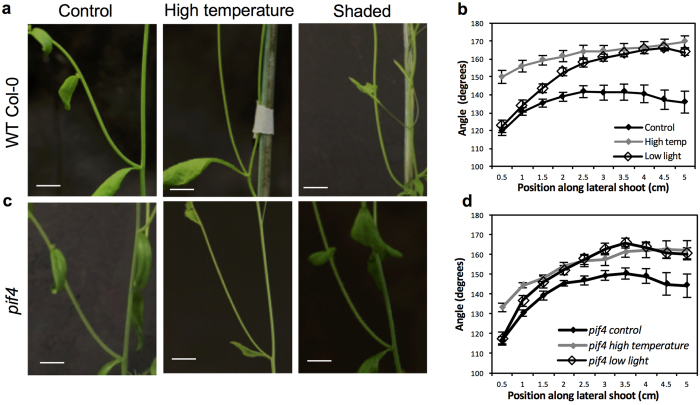
Effects of temperature and light on shoot branch GSA in Arabidopsis. High temperature and low light/low R/FR conditions induce a significantly more vertical lateral branch GSA wild-type Col-0 (**a,b**) and *pif4-2* mutant plants (**c,d**). Scale bar = 1 cm. Bars represent s.e.m of 6-10 shoots each from three independent experiments.

In order to explore how environmental cues could affect shoot branch GSA, we examined the effect of light and temperature on Arabidopsis cauline branch GSA. During the course of the experiment, plants were grown under conditions of lower light intensity and low red/far-red (R/FR) light ratios (20 μmol m^−2 ^s^−1^;R/FR = 0.3; 20 °C) or high temperature (200 μmol m^−2 ^s^−1^;R/FR = 1;28 °C). Under both regimes plants displayed features characteristic of the classic shade avoidance response such as increased hypocotyl length, more vertical rosette leaf inclination angle and early bolting (see Casal, 2013^8^ for review) compared to plants grown in unshaded control conditions (200 μmol m^−2 ^s^−1^;R/FR = 1;20 °C). This was accompanied by plants grown in shaded and high temperature conditions displaying significantly more vertical lateral branch GSAs than those grown in the control conditions (Fig. 5a,b).

Previous work has shown that the growth phenotypes induced at high temperatures and during vegetational shade both involve the upregulation of auxin biosynthesis and are mediated by the bLHL transcriptional regulator PHYTOCHROME INTERACTING FACTOR 4 (PIF4)^13^,^32^,^33^. We therefore decided to investigate the effect of variation in light or temperature conditions on the lateral branch GSA of the *pif4–2* mutant. As expected, *pif4-2* mutant seedlings grown under low R/FR conditions and high temperature conditions did not display shade avoidance or high temperature related phenotypes such as elongated hypocotyls or early flowering (Fig. S4a–c). Also, the lateral branch GSA of *pif4-2* plants was slightly, but not significantly more vertical than that of wild-type Col-0 under control conditions (Fig. S4d). However, after bolting, *pif4-2* mutant plants displayed more vertical GSA phenotypes in both low R:FR and high temperature conditions (Fig. 5c,d). These data suggest that while other classic shade avoidance and high temperature phenotypes may be mediated by PIF4-dependent auxin biosynthesis, the environmental regulation of lateral branch GSA occurs through a PIF4-independent pathway.

## Discussion

### Auxin as a nexus for the developmental and environmental control of branch GSA

The central role of auxin in lateral branch GSA control provided a basis for exploring the developmental regulation of branch GSA and also its modulation by environmental signals. Increases in endogenous auxin levels or treatment with exogenous auxin causes lateral root and shoot branches to grow at more vertical GSAs^6^. Using the Arabidopsis lateral root as a model we have shown here that the increasingly vertical pattern of growth of lateral roots is accompanied by an apparent increase in endogenous auxin levels as the lateral root grows out from the main root. These experiments used the Aux/IAA-based reporters DII-Venus and R2D2 the auxin-induced degradation of which is dependent on TIR1/AFB auxin co-receptor proteins. The analysis of the expression of TIR1, AFB2, and AFB3 using translational markers allowed us to test for potential differences in the capacity to perceive auxin between younger and older lateral roots. Because Arabidopsis mutants and transgenic lines with increased auxin sensitivity have been shown to cause lateral roots to grow at more vertical GSAs^6^, any such differences in TIR1/AFB expression might have been able to account for at least some of the GSA variation observed during lateral root development. The lack of significant differences in co-receptor levels between younger and older lateral roots indicates that in Arabidopsis, gross variation in TIR1/AFB expression is not a component of the developmental control of lateral root GSA. These experiments nevertheless provided important validation for the use of DII-Venus and R2D2 to infer auxin levels in Arabidopsis lateral root tips.

### Nitrate and phosphate deficiency have opposite effects on the GSA of lateral roots in Arabidopsis and basal roots in bean

Phosphate deficiency has previously been shown to effect marked changes in root system architecture including the shortening of the primary root and an increase in the formation and emergence of lateral roots^19^,^20^,^21^. In the present study, phosphate deficiency also caused a significant shift in lateral root GSA towards a more vertical orientation, confirming the prior work of Bai *et al*.^24^. Previous studies measured free IAA levels in roots of phosphorus-replete and phosphorus-deprived plants and found that while auxin levels remain the same, increased *TIR1* expression causes cells to become more responsive to endogenous auxin concentrations^20^,^30^. This work is supported by several studies that showed that the roots of phosphate-deprived seedlings are more responsive to exogenous auxins than those of seedlings grown in phosphorous-replete conditions^22^,^34^. Thus, the more vertical lateral root GSA phenotype seen in phosphate-deprived seedlings is consistent with both these findings and our previous work on the central importance of transcriptional responses to auxin in GSA control^6^. However, in the work presented here, the low phosphate effect on lateral root GSA in the *tir1/afb* single loss-of-function mutants was found to be of similar magnitude to that observed in wild-type, albeit shifted to a less vertical GSA range as previously reported for the *tir1-1* mutant^6^. The significant increases in both *TIR1* and *AFB3* transcriptional and translational markers in response to low phosphate suggested possible redundancy between these auxin co-receptor proteins. Indeed, loss-of-function of both genes in the *tir1 afb3* double mutant abolished the lateral root GSA response to phosphorous deficiency, confirming that redundantly-acting TIR1 and/or AFB3 are required to mediate the low phosphate response.

In contrast to phosphate deprivation, nitrate starvation resulted in a more horizontal lateral root GSA in Arabidopsis. Several root architectural changes in nitrate-deficient conditions have been shown to be mediated through a *miR393/AFB3* regulatory module in Arabidopsis^18^ and indeed *afb3-4* mutant seedlings failed to respond to nitrate induced lateral root GSA modulation. These data suggest that in contrast to low phosphate responses, the effects of nitrate deficiency on root system architecture may be mediated through a non-redundant AFB3 signalling module.

### Basal root GSA in bean

Several previous studies have shown that maize, bean and soybean varieties with shallower root growth angle have up to a six-fold greater capacity to forage topsoil and therefore increase phosphate acquisition compared to deeper-rooting varieties^35^,^36^,^37^. This is because phosphate is relatively immobile in soil meaning that upper soil horizons are phosphate-enriched because of the decomposition of plant and other organic matter^38^. Thus the change in basal/adventitious root GSA to a less vertical orientation in phosphorus-deficient conditions would appear to reflect an adaptation to enhance increase phosphate acquisition. The low phosphate-induced shift in GSA observed in bean basal roots is opposite to that seen in Arabidopsis lateral roots. Indeed, treatment with low concentrations of IAA (50–70 nM) is sufficient to induce less vertical growth in bean basal roots and more vertical growth in bean and Arabidopsis lateral roots^6^ indicating that these contrasting responses to low phosphate might share a common auxin-related mechanism. Treatment with higher auxin concentrations (90–100 nM IAA) caused bean basal roots to also show more vertical growth suggesting that there is differential sensitivity to auxin between these classes of root branches. To some extent these differences in growth angle response to auxin between adventitious and lateral roots mirror the opposing cell elongation responses to auxin typically observed in roots and shoots. Thus, if the contrasting root growth angle responses are determined at the level of the expanding cells in the root elongation zone, it may be pertinent to explore the possible significance of the fact that adventitious roots, by definition, arise from shoot rather than root tissues. Further work will be required to determine the physiological basis of these interesting differences in growth angle control between adventitious and lateral roots.

### Low light levels and R/FR ratios cause Arabidopsis shoot branches to shift towards a more vertical GSA

Among the diverse external environmental stimuli perceived by plants, light is one of the most important. In Arabidopsis, light is able to influence multiple facets of the auxin system, controlling auxin levels, transport, and responsiveness (Reviewed in ref. 39,40). Previous studies have shown that phytochrome light receptors have a strong influence on auxin homeostasis in plants by regulating both *SUR2*, a repressor^41^ and *TAA1*, an enhancer of auxin biosynthesis^10^. When R/FR ratios are high, active PhyB reduces IAA levels by coordinated activation of SUR2, and reduction of *TAA1* transcript levels. Conversely, reduced levels of PhyB, induced by lower R/FR ratios trigger reciprocal regulation, with a consequential increase in IAA levels. These changes in auxin levels have been shown to be central to the shade avoidance response. In higher plants, the shade avoidance syndrome (SAS) consists of a series of responses to low light levels, the most dramatic of which is rapid increased elongation of internodes, hypocotyls and petioles. Additionally, vegetational shade leads to accelerated flowering times, reduction in leaf size, and leaf hyponasty^8^. Previous work has shown that the responses induced during vegetational shading involve the upregulation of auxin biosynthesis and are commonly mediated by the bLHL transcriptional regulator PHYTOCHROME INTERACTING FACTOR 4 (PIF4)^13^,^33^ that directly activates the expression of *YUC8*, a member of the YUCCA auxin biosynthesis gene family and therefore elevates free IAA levels^42^. In addition, light also modulates transcript levels of the *PIN3* auxin efflux transporter: activation of PhyB reduces *PIN3* transcript abundance, while PhyB inactivation resulted in increased *PIN3* transcript levels^11^. Further, Laxmi *et al*.^43^ showed that light signalling could modulate auxin flux by directly regulating the intracellular localization of PIN1, PIN2 and PIN7 proteins. These results along with work from Friml *et al*.^44^ showing light-dependent relocation of PIN3 in Arabidopsis hypocotyls suggest that light is able to modulate auxin transport by regulating intracellular PIN distribution.

In the present study, it was found that lower R/FR ratios and light intensity resulting from artificial vegetational shading induce a more vertical GSA phenotype in Arabidopsis shoot branches. This response is compatible with the suite of developmental changes characteristic of the shade avoidance syndrome in that it contributes to limiting lateral growth towards neighbouring vegetation where shading is likely to be encountered. At the mechanistic level, the increased verticality of branches is also consistent with both the work described above where decreases in R/FR ratios lead to TAA1-mediated increases in auxin biosynthesis^10^ and our previous work on GSA control in which we demonstrated that increases in endogenous auxin levels causes lateral branches in Arabidopsis to adopt more vertical GSAs^6^. Interestingly, a recent study^9^ also showed that low R:FR ratios result in increased auxin sensitivity in Arabdiopsis providing a possible additional input into light quality-regulated GSA control. Together, these findings indicate that more vertical branch growth angle in Arabidopsis should be considered to be a functionally important component of the shade avoidance syndrome.

### High temperature causes Arabidopsis shoot branches to shift towards a more vertical GSA

Temperature as an environmental cue has long been known to have a dramatic effect on plant architecture by inducing rapid hypocotyl elongation, leaf hyponasty, and early flowering^12^,^13^,^14^. In this study, high temperature was also found to cause a shift in lateral branch GSA towards a more vertical orientation. This is consistent with early work which revealed a correlation between high temperature-induced hypocotyl elongation was associated with elevated free IAA levels in Arabidopsis^12^. Genetic analysis revealed that high temperature-induced hypocotyl elongation was drastically reduced in Arabidopsis mutants defective in auxin biosynthesis, signalling or transport, confirming a central role of the auxin signalling pathway in mediating high temperature induced architectural changes in Arabidopsis. More recently, PIF4, a member of the phytochrome interacting PHYTOCHROME INTERACTING FACTOR family of proteins (the PIFs) that are known to be versatile integrators of environmental and hormonal signalling pathways, has been shown to be a key mediator of high temperature-induced hypocotyl elongation regulating auxin biosynthesis. In response to high temperature, PIF4 was found to directly activate the expression of *YUC8* and so elevate free IAA levels^33^. Moreover, *pif4-2* mutants largely lost the robust enhancement of hypocotyl elongation induced by high temperature^13^. Interestingly, while *pif4-2* seedlings did not have elongated hypocotyls or early flowering responses in low light or high temperature conditions, the lateral branches of the *pif4-2* mutant displayed a more vertical growth regime under these conditions similar to that observed in wild-type plants. These data suggest that in Arabidopsis, many but not all architectural changes in response to low light and temperature are integrated at the level of a PIF4-dependent signalling pathway. Indeed, this finding is consistent with recent work showing that in addition to PIF4-mediated auxin accumulation, high temperature response in Arabidopsis also includes the stabilisation of the auxin co-receptor TIR1, a mechanism that is dependent on chaperone protein HSP90^14^. In keeping with our previous work on the TIR1/AFB-dependent control of GSA^6^, this increase in TIR1 levels would be predicted to cause shoot branches to grow at more vertical GSAs.

## Conclusions

Previous work has shown that the plant hormone auxin controls lateral organ GSA in Arabidopsis, and that spatial variation in auxin concentration and/or sensitivity is sufficient to regulate GSA. The data presented here suggest that environmental signals such as light, temperature and nutrient availability are able to effect changes in both lateral root and shoot GSA through the modulation of TIR1-dependent auxin signalling and consequent changes in auxin levels and/or sensitivity. The integration of intrinsic developmental and extrinsic environmental signals into the canonical auxin signalling pathway thus provides an important regulatory mechanism by which plants can modify root and shoot system architecture to enhance or alter resource acquisition in changing environmental conditions.

In the context of opportunities for the optimisation of root and shoot system function for crop improvement via the manipulation of growth angle there are important considerations arising from this work. In particular, the contrast between the response to low phosphate of basal roots in bean, and lateral roots of Arabidopsis and bean highlights the fact that not all changes in plant architecture in response to environmental stresses will be optimal with respect to those stresses. The responses to phosphate deprivation in bean and nitrate deprivation in maize^45^,^46^,^47^ provide a useful example. In Arabidopsis we, and previous researchers^24^, found that low phosphate induced a more vertical lateral root GSA. This is a developmental response which at first sight is at odds with the extensive work from the Lynch Lab at Penn State showing that shallower rooting systems provide a significant improvement in performance on phosphate poor soils^31^,^35^,^36^,^45^. Most of this work was done in the common bean and indeed this was the motivation for looking at the effect of auxin on bean root GSA. Understanding why low levels of exogenous auxin have opposite effects on growth angle in bean and Arabidopsis lateral roots, and bean adventitious roots (which, as noted above, originate from different parent tissues) will be an interesting phenomenon to explore. Similarly, it has been proposed that in maize, the ideotype for low nitrogen soils would include more vertical root growth^46^ which, again, is the opposite response to that observed in Arabidopsis root systems grown at low nitrogen. Therefore, assuming that these strategies to enhance nutrient aquisition in bean and maize are broadly applicable then from an evolutionary developmental biology viewpoint, and for the rather small weed Arabidopsis at least, the lateral root GSA responses to low phosphorous and low nitrogen must be considered as less critical to plant fitness than the basic mechanism of GSA control that permits the formation of a laterally expanded root system to begin with. Importantly, the deeper understanding of the mechanistic basis of GSA regulation that has developed over recent years provides a set of tools and approaches allowing the distinct developmental processes regulating root system architecture to be uncoupled and modulated independently of one another.

## Methods:

### Plant materials

All Arabidopsis seed stocks are in the Col-0 background unless otherwise stated. R2D2 is in the Utrecht background^26^. Seeds of *tir1-1*^48^, *afb1-3, afb3-4, tir1-1 afb3-4* and *TIR1/*AFB transcriptional fusion marker lines^29^ were kindly provided by Prof. Mark Estelle, while *pif4-2*^49^ was obtained from the Nottingham Arabidopsis Stock Centre. Common bean (*Phaseolus vulgaris*) seeds were obtained from Wilkinson Hardware Stores, Worksop, UK.

### Construction of TIR1/AFB fluorescent marker lines

The *pTIR1:gTIR1-Venus* and *pAFB2:gAFB2-Venus* lines have previously been described^14^. *pAFB3:AFB3-Venus* was constructed with the same approach: a genomic fragment consisting of 4-kb of promoter and the coding sequence of the gene, fused in frame with the coding sequence for Venus, followed by 2-kb of 3′ sequence. Traditional cloning strategies including PCR, endonuclease digestion, and ligation were adopted to introduce the fragments into the binary vector pGREENII 0229^50^. Details of the primers used for cloning are shown in supplementary Table 1. The resulting *pAFB3:gAFB3-VENUS* was used to transform *afb3-4*^29^.

### qRT-PCR for TIR1 expression

RNA was extracted from the root systems of 12 day-old wild-type Col-0 plants grown in phosphorus-replete and phosphorus-deficient conditions using the Qiagen RNAeasy kit according to the manufacturer’s instructions. cDNA was synthesized from the isolated RNA using oligo dT primers and Superscript II reverse transcriptase (Invitrogen). qPCR was performed using the Bio-Rad CFX Connect Real-Time System (Bio-Rad). EF1α was used as an internal control.

### Confocal microscopy

10–12 day old DII-Venus, R2D2 and TIR1/AFB marker seedlings grown in phosphorous-replete or -deficient conditions were imaged at 20X resolution with the 480 nm and 540 nm lasers using a Zeiss LSM 700 inverted confocal microscope. DII-Venus seedlings were counterstained with propoidium iodide prior to imaging. All laser power and gain settings were consistent across images. Fluorescent intensity was quantified using ImageJ. Excluding the lateral root cap, nuclear fluorescence was measured in ten consecutive epidermal cells within the two outermost flanking cell files, beginning from the root tip for each root. At least ten root tips were used for each experiment. For R2D2, nuclear fluorescence intensity was measured across both GFP and mTomato channels. For each nucleus, the ratio of GFP/mTomato signal was determined. Geometric means and standard errors of the ratios were calculated for both young and older lateral roots. Student’s T-test was performed to evaluate statistical differences between the geometric means of the data obtained.

### GUS staining

TIR1/AFB transcriptional marker lines were grown in phosphorus-replete and phosphorus-deficient conditions. 12 days post germination, intact seedlings were subjected to GUS staining using the protocol described in Vitha *et al*.^51^. Seedling lateral roots were imaged using an Olympus SZX9 light microscope.

### Analysis of lateral root GSA

For lateral root GSA measurements under high and low nitrate conditions, 12 day old Arabidopsis seedlings were grown on 120 mm square petri plates with modified *Arabidopsis thaliana* salts (ATS) medium^6^ in standard tissue culture conditions (18–20 °C, 16 h day and 8 h dark cycles). For both high and low nitrate conditions, 2 mM CaCl_2_ was used as a substitute for 2 mM Ca(NO_3_)_2_. For the high nitrate treatment KNO_3_ was kept at 5 mM while for the low nitrate treatment the concentration of KNO_3_ was reduced to 0.1 mM. For low phosphate experiments, seeds were grown on Johnson’s medium (1 mM Ca(NO_3_)_2_, 2 mM KNO_3_, 1 mM NH_4_NO_3_, 0.5 mM MgSO_4_, 1 mM KCl, 2 mM K_2_SO_4_, 1 M CaCl_2_, 250 mM MES, 20 μM FeNa_2_EDTA, 0.1 mM H_3_BO_3_, 0.22 μM CoCl_2_, 0.1 μM CuCl_2_, 0.1 mM MnCl_2_, 0.1 μM NaMoO_4_, 5 μM NaI, 0.05 mM ZnCl_2_ with either 500 μM KH_2_PO_4_/K_2_HPO_4_ for high phosphate or 0 μM KH_2_PO_4_/K_2_HPO_4_ for low phosphate conditions with 8 g/L washed agar^52^. The plates were scanned using a HP Scanjet G4050 photo scanner and the images obtained were analysed using ImageJ. Each lateral root analysed was divided into 0.5 mm segments and the growth angle of each segment was measured with reference to the gravity vector. The data was statistically evaluated using the Wilkes-Shapiro and Kolmogorov-Shapiro tests for normality followed by a paired t-test or one-way ANOVA.

### Analysis of lateral shoot GSA

To analyse lateral shoot GSA, seeds of various plant lines were sown in small 5 cm pots containing compost which were stratified for 48 hours to promote uniform germination. After germination, seedlings were transplanted to individual square pots and allowed to grow for 28 days in the greenhouse at a photoperiod of 16 h day and 8 h dark cycles. Control conditions consisted of a temperature of 20 °C and 200 μmol m^−2 ^s^−1^ light intensity, with red/far red ratios approximately equal to 1. For high temperature conditions, plants were grown at 28 °C with similar light conditions. For low light intensity conditions, green shading netting (LBS Horticulture, Lancashire) was used to reduce the light intensity to 20 μmol m^−2 ^s^−1^, with a red/far red ratio of approximately 0.3. For low light and high temperature conditions, often the primary inflorescence stem was not supported by the lower rosette leaves of the plants. In these cases, the primary stem was restricted at a vertical orientation using a thin stake. Photographs of individual branches were taken using a Canon G9 digital camera, and the GSA of individual lateral branches was measured using ImageJ. Each shoot was divided into 0.5 cm segments and the growth angle of each segment was measured with reference to the gravity vector. The data were evaluated using the statistical tests described above. For *pif4-2* phenotyping experiments, plants were grown in control, high temperature, and shaded conditions and photographed after 14 days using a Sony RX100 digital camera. For *pif4-2* hypocotyl experiments, sterile *pif4-2* and Col-0 seeds were sowed on 120 mm square ATS plates and placed in a vertical orientation in light or shaded conditions. Hypocotyl length was measured after 12 days.

### Bean basal root experiments

For bean experiments, seeds were surface sterilised using chlorine gas and germinated in a petri plate containing moistened filter paper. After germination, seeds were transferred into individual cyg^TM^ seed germination pouches (Mega International, Minnesota, USA) containing 50 ml of nutrient medium previously described in Bonser *et al*.^31^, with 50 or 100 nM IAA for hormone treatments. The seedlings were allowed to grow in pouches for seven days prior to being photographed using a Canon G9 digital camera. The growth angle of each basal root generated by a 1 cm segment of root from its point of origin was measured relative to the vertical, again, using ImageJ. Similarly, for lateral roots, the growth angle of each lateral root generated by a 1 cm segment of root from its point of origin was measured relative to the vertical. Statistical analysis was performed using one way ANOVA, followed by posthoc Tukey’s Honestly Significant Difference test.

## Additional Information

**How to cite this article**: Roychoudhry, S. *et al*. The developmental and environmental regulation of gravitropic setpoint angle in Arabidopsis and bean. *Sci. Rep.* **7**, 42664; doi: 10.1038/srep42664 (2017).

**Publisher's note:** Springer Nature remains neutral with regard to jurisdictional claims in published maps and institutional affiliations.

## Supplementary Material

Supplementary Information
