# Supplementary material for: The developmental and environmental regulation of gravitropic setpoint angle in Arabidopsis and bean
Source: Sci Rep. 2017 Mar 3;7:42664. doi: 10.1038/srep42664 (PMC5335621; doi:10.1038/srep42664)
Supplement: Supplementary Information [file srep42664-s1.pdf]

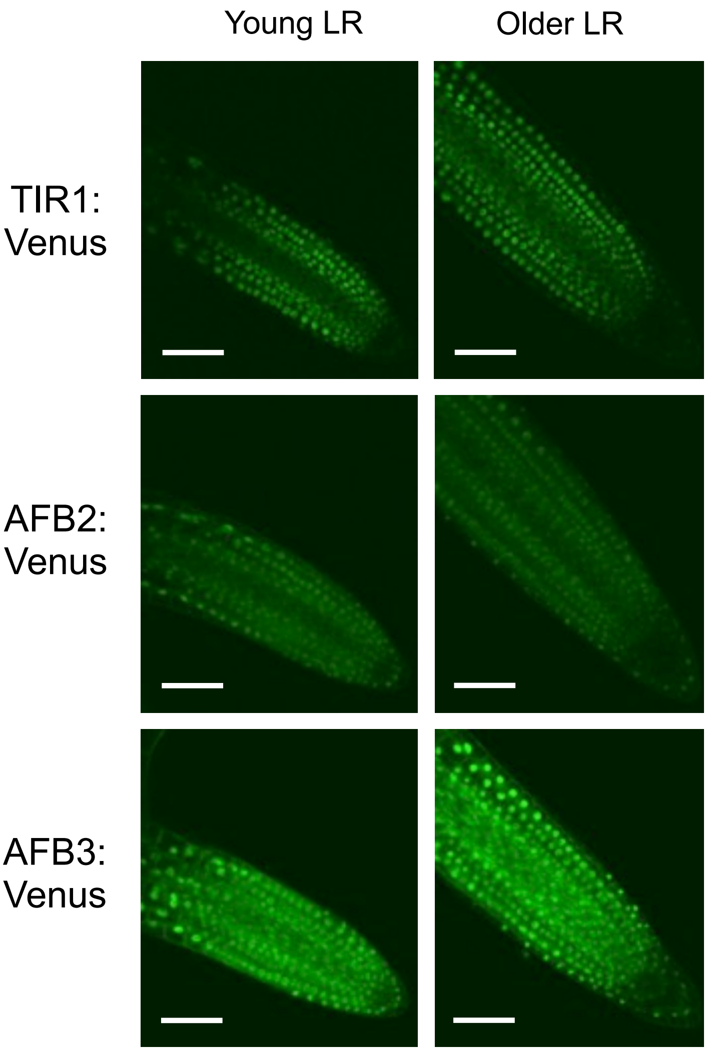

Supplementary Figure 1. Comparative expression of TIR1/AFB receptor translational reporter lines in young and old lateral roots in Arabidopsis. No significant differences in expression were seen in lateral roots of different ages. *pTIR1::TIR1:Venus* (TIR1:Venus); *pAFB2::AFB2:Venus* (AFB2:Venus); *pAFB3::AFB3:Venus* (AFB3:Venus). Scale bar = 30 μM.

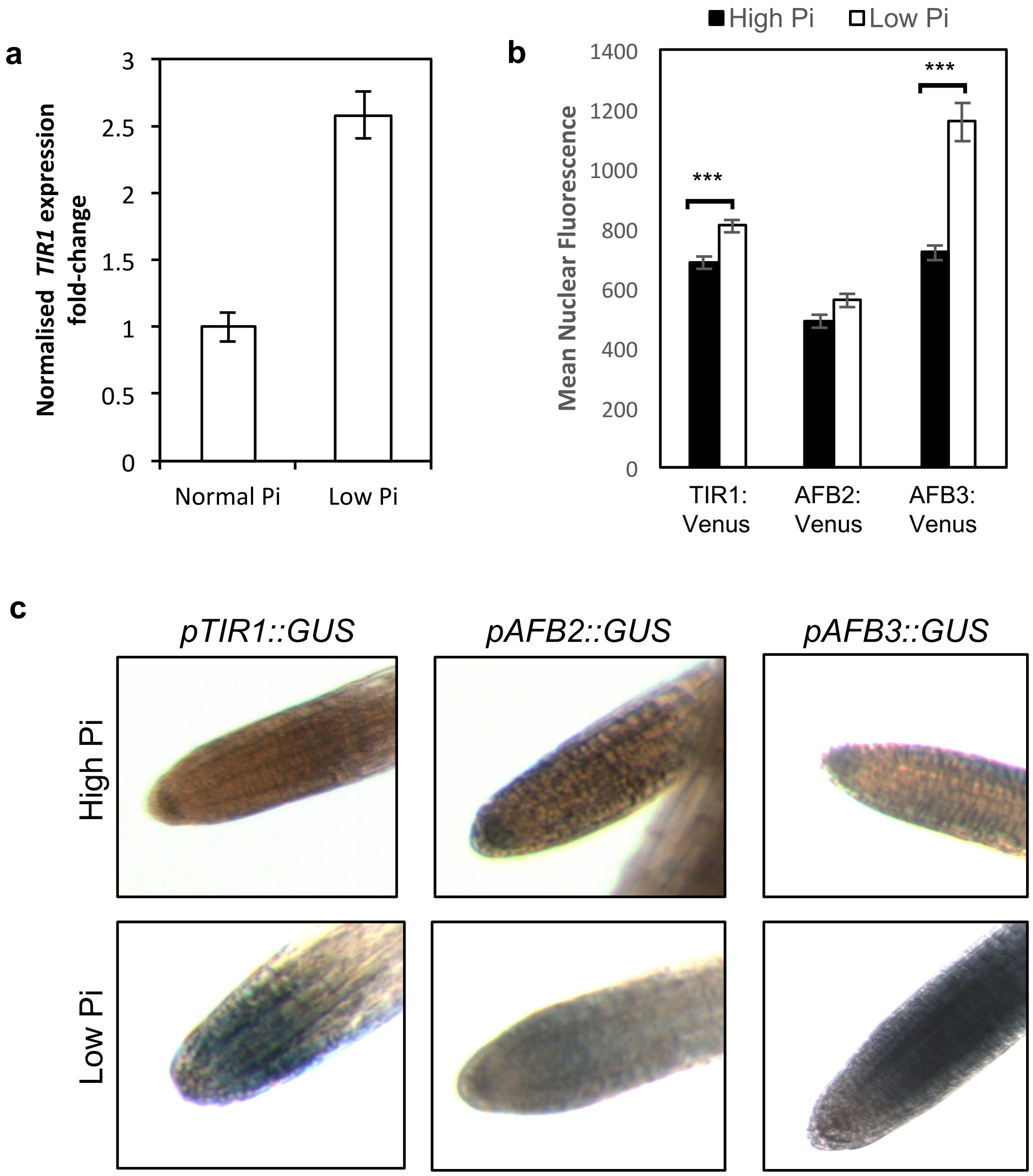

Supplementary Figure 2. ***TIR1/AFB*** **expression under phosphorus replete and –deficient conditions.** *TIR1* expression increases approximately 2.5 fold in roots of wild-type Arabidopsis seedlings grown in phosphorus-deficient conditions (**a**). Phosphorus deficiency induces significant increases in TIR1:Venus (*pTIR1::TIR1:VENUS)* and AFB3:Venus (*pAFB3::AFB3:VENUS)* levels in Arabidopsis lateral roots (**b**). Students T-tests gave p-values of 2.77×10^{-5} for TIR1, and 1.65×10^{-10} for AFB3. $p > 0.05$ for AFB2. X-Gluc staining of lateral roots of *pTIR1/AFB::GUS* transcriptional marker lines grown in phosphorus-replete and phosphorus-deficient conditions (**c**). Bars represent s.e.m.

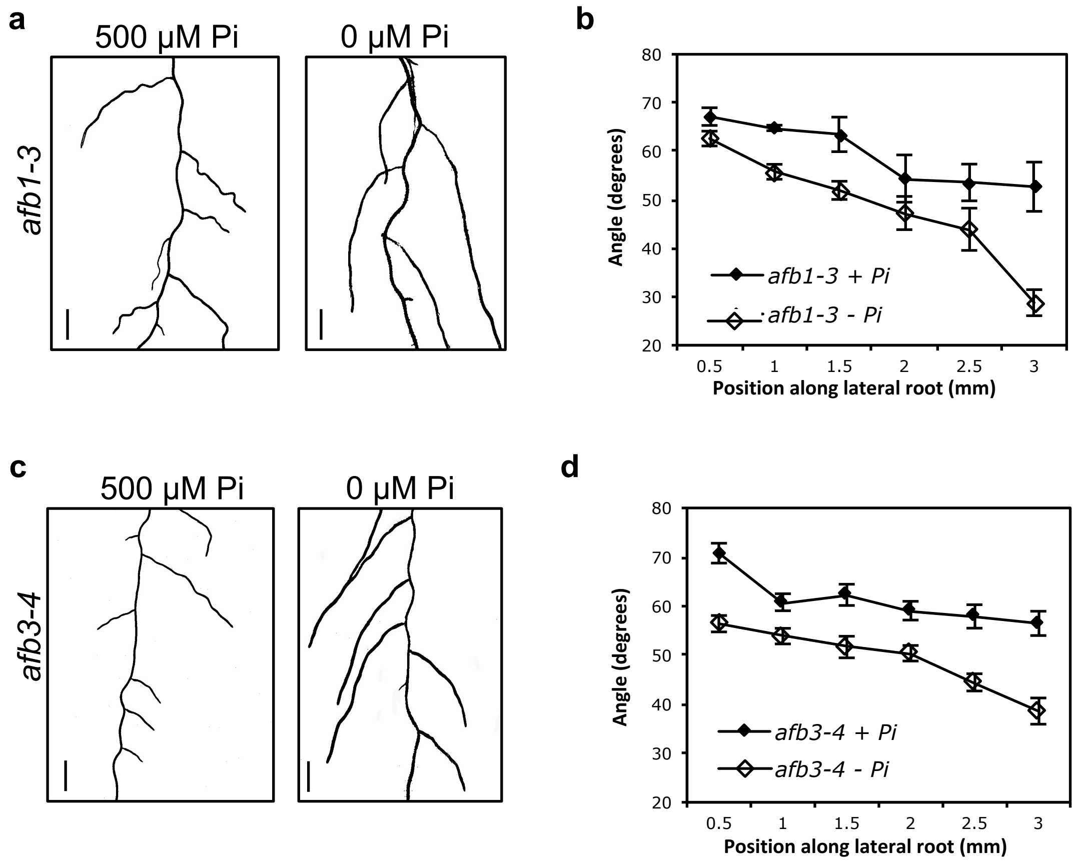

Supplementary Figure 3. Effect of phosphate deficiency on lateral root GSA in Arabidopsis Low phosphorus levels cause lateral root GSA in *afb1-3* (**a**,**b**) and *afb3-4* (**c**,**d**) mutant seedlings to shift towards a more vertical GSA. Scale = 5 mm. Bars represent s.e.m.

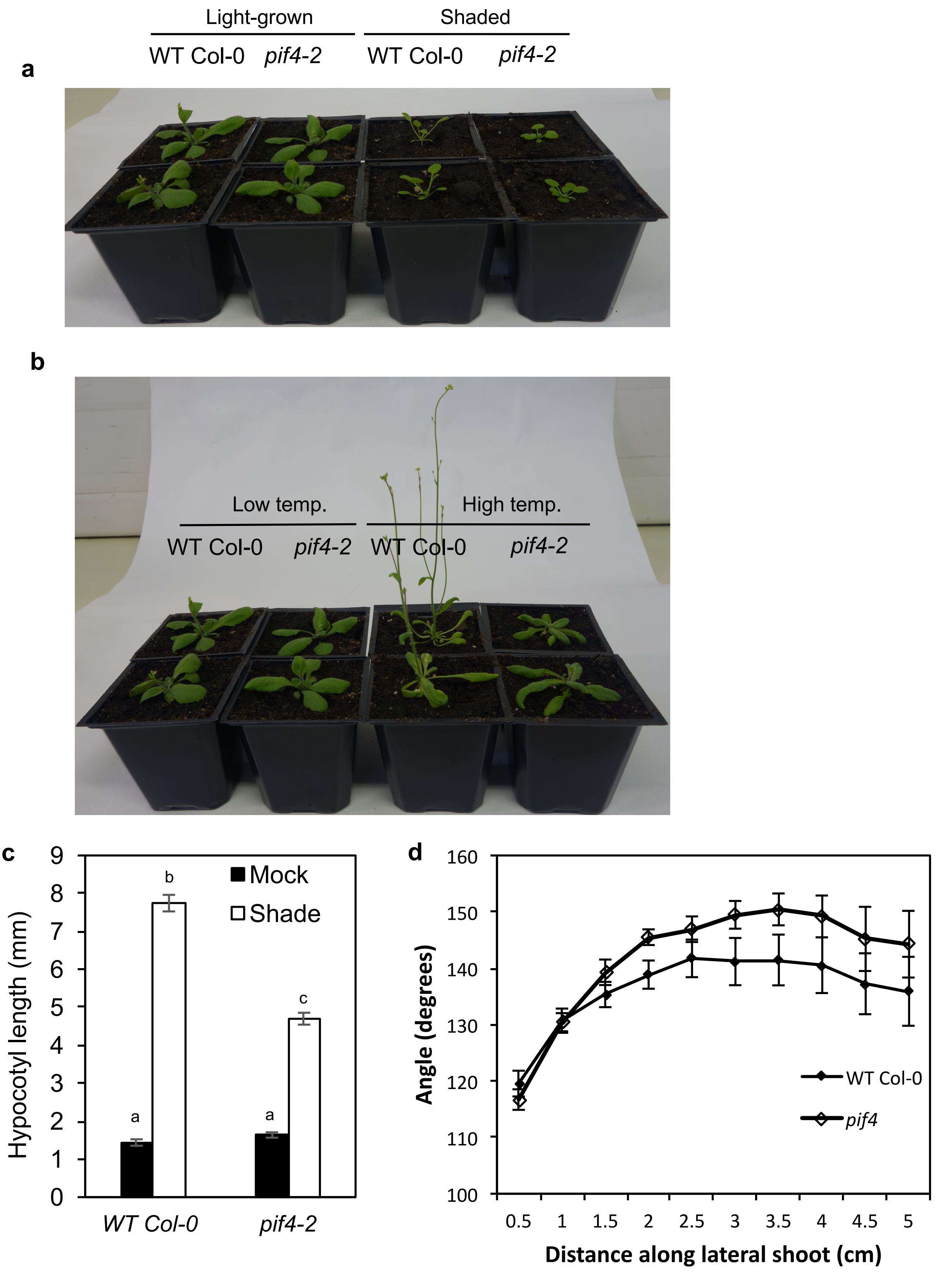

Supplementary Figure 4. Effects of temperature and light on shoot branch GSA in Arabidopsis Phenotypes of WT Col-0 and *pif4-2* adult plants under (**a**) shaded and (**b**) high temperature conditions. (**c**) Hypocotyls of WT Col-0 seedlings elongate significantly more than those of *pif4-2* under shaded conditions. Different letters indicate significant differences at $p < 0.01$. (**d**) Quantification of lateral branch GSAs of wild-type Col-0 and *pif4-2* mutant plants. *pif4-2* lateral branches are slightly but not significantly more vertical than those of Col-0.
